# Enhancing pH prediction accuracy in Al_2_O_3_ gated ISFET using XGBoost regressor and stacking ensemble learning

**DOI:** 10.1038/s41598-025-04530-2

**Published:** 2025-06-01

**Authors:** Ashirbad Panda, Rishikesh Datar, Shreyas Deshpande, Gautam Bacher

**Affiliations:** Department of Electrical and Electronics Engineering, BITS Pilani K K Birla Goa Campus, Goa, 403726 India

**Keywords:** Al_2_O_3_, Hyperparameter optimization, ISFET, Regressor, Stacking ensemble learning, XGBoost, Biomedical engineering, Predictive markers

## Abstract

An ion-sensitive field-effect transistor (ISFET) is widely used in environmental and biomedical applications due to its rapid response, miniaturization, and cost-effectiveness. In this study, a numerical model of an Al₂O₃-gated ISFET was developed to detect pH levels. The effects of gate dielectric thickness, doping concentration, and temperature on ISFET’s performance were evaluated using I_DS_–V_DS_ characteristics. An eXtreme Gradient Boosting (XGBoost) regression model was employed to predict pH levels using data obtained from I_DS_–V_DS_ characteristics. Further, Hyperparameter optimization was performed to tune critical XGBoost-hyperparameters such as maximum depth, minimum child weight, estimators, learning rate, α, and λ. The optimization strategies such as random search, grid search and Bayesian optimization were utilized to improve the efficacy of regressor by minimizing errors and maximizing accuracy in prediction. A stacking ensemble learning approach was also implemented to integrate multiple models, enhancing prediction accuracy and thereby capturing additional information. The XGBoost regressor achieved superior results with R^2^ = 0.9846, MSE = 0.2342, and MAE = 0.2317, compared to other regressor models. Therefore, the use of XGBoost regressors with hyperparameter optimization and stacking ensemble learning approach is found to be highly effective for pH prediction from ISFET under various operating conditions.

**Supplementary Information:**

The online version contains supplementary material available at 10.1038/s41598-025-04530-2.

## Introduction

Ion-sensitive field-effect transistors (ISFETs) are electrochemical sensors that employ a field-effect mechanism to transform ion concentrations into equivalent electrical signals. ISFETs have attracted significant attention in chemical and biosensing applications owing to their high sensitivity, robust sensing, compatibility with fabrication processes and low cost^1^. It resembles a metal-oxide semiconductor field-effect transistor (MOSFET), but incorporates an insulator/electrolyte interface and a reference electrode immersed in an aqueous solution. ISFET uses solid-state technology and is compatible with CMOS technology. Additionally, it enables to develop of miniatured and low-power devices that are suitable for point-of-care applications. The sensing mechanism involves site binding activity at the insulator/electrolyte interface of ISFET, generating an electric double layer. This phenomenon directly affects threshold voltage (V_TH_) and drain-to-source current (I_DS_)^2^. However, the performance of ISFET may deteriorate due to offset, flicker noise, drift, degradation in structural morphology of gate-dielectrics and non-linear temperature-related properties^3^. The mathematical model of ISFET helps in obtaining the intended output in terms of I-V characteristics of ISFET. These models are often used to assess the influence of several geometrical, physical, material, chemical, and operational parameters on ISFET performance^4,5^. Several studies have reported an investigation of ISFET models using high-K dielectric materials and various FET configurations, such as fully-depleted silicon-on-insulator (FDSOI), tunnel, and nanowire-based ISFETs, using appropriate computational tools^6,7^. Rasool et al. have modelled the doping-less tunnel FET-based pH sensor in an aqueous electrolyte environment by enhancing sensitivity using Al_2_O_3_ as a sensitive oxide layer^8^. The high-K dielectric materials, such as Si_3_N_4_, Al_2_O_3_, Ta_2_O_5_, and HfO_2_, are reported in ISFET-based sensors to enhance sensitivity^9–11^. This gate dielectric material of ISFET plays a key role in achieving near-Nernstian (~ 59 mV/pH) or super-Nernstian (> 59 mV/pH) voltage sensitivity in pH sensing. However, ISFET remains in continuous contact with the electrolyte, which may lead to the degradation of the sensing gate-dielectric layer over time^12^. Our previous work demonstrated that persistent use of ISFET in sensing leads to a reduction in gate insulator thickness, hence impacting the dielectric properties of the material and its capacitance^4^. ISFET performance is also influenced by the geometrical changes, doping profile and temperature, potentially leading to erroneous results. In these events, the analytical and numerical modelling techniques are effective in incorporating or recreating the undesirable changes in ISFET model using suitable tool. Therefore, a computationally efficient method is essential for detecting parameter shifts during sensing and correcting the ISFET output accordingly to the undesired changes.

Generally, a precise detection of the analyte in bio/chemical sensing is critical due to operational and measurement variability of the device. The errors in the measurements can potentially be addressed by predicting the accurate response of the sensor by machine learning (ML). ML-assisted sensor exhibits a high degree of precision and occasionally eliminates the need for expert involvement. Moreover, ML techniques facilitate to train, analyse the data, predict the sensitivities, extract the sensor responses, and calibration of the sensor^13,14^. Nowadays, the ML-based approach has emerged as a prominent and impactful tool to train the extensive datasets of electrical and electrochemical sensors^15^. There have been substantial developments in ML for various sensing applications in the domain of healthcare, environmental monitoring, food security, and agriculture^16^. ML-assisted bio/chemical sensors have been demonstrated in improvement of sensing performance and their interpretability of the data gathered^17^. Recently, ISFET with advanced ML techniques has shown great promise to improve the sensing accuracy, reliability, and adaptability of measurements^18^.

Several ML techniques, including k-nearest neighbour (k-NN), neural networks (NN), support vector machines (SVM), and decision trees, have become significant for electrochemical sensing^15^. Pal et al. developed a robust ML classifier for precise detection of endometriosis using an ensemble bagged tree model^19^. Moreover, the effective area of different biomembranes was detected using electrochemical impedance spectra based on support vector regression by predicting electrode diameter^20^. Sahu et al. have developed recurrent neural networks and multilayer perceptron models to compensate for the temporal and temperature drift in ISFET-based pH sensors using root mean squared error^21^. Moparthi et al. have predicted the influence of critical device characteristics, including core radius and channel length, on the performance of silicon nanotube FET via random forest and gradient boosting regression techniques^22^. In addition, some ML techniques exhibit several limitations depending on the dataset and intended application. Linear regression is inappropriate for complex relationships, while k-NN is sensitive to irrelevant features^23^. The neural network technique demands extensive datasets and is computationally intensive. However, gradient boosting is a sophisticated method designed to enhance speed and performance in predictive modelling. eXtreme gradient boosting decision tree (XGBoost) algorithm serves as a prominent tree-based ensemble model that enhances the prediction ability of decision trees by focusing on the residual errors of prior classifiers^24^. Furthermore, it can handle large datasets with high dimensionality, making it particularly suitable for bio/chemical sensing. There is limited study on ML techniques using XGBoost algorithm for precise pH prediction based on the complex input–output relationship in ISFETs.

Moreover, the trained ML model can attain efficiency, accuracy, and reliability through effective hyperparameter selection and tuning. Hyperparameter optimization (HPO) is essential for regulating the learning process and enhancing performance. Hyperparameters are the external configuration parameters of a model that need to be defined prior to the learning phase to minimize the objective function^25,26^. Choosing the optimal set of hyperparameters for ML techniques has a direct influence on model performance, training efficiency, robustness, and to automate the search^27^. It ensures that models are precise, stable, and computationally efficient, resulting in superior and more reliable outcomes. Prevalent HPO techniques include grid search (GS), random search (RS), and Bayesian optimization (BO). GS exhaustively evaluates each possible hyperparameter combination, proving it essential but time-consuming and impractical for extensive datasets. RS mitigates the dimensionality constraints of GS by randomly selecting hyperparameter values. However, it is inefficient since it fails to leverage prior evaluations. Bayesian optimization is a probabilistic approach used to identify the minimum of a function, with the goal to find the input value that produces the lowest output^28^. It effectively balances the exploration and exploitation to determine optimum hyperparameters. It utilizes a surrogate model, such as a Gaussian process, to characterize the objective function. It applies to acquisition functions like Thompson sampling or expected improvement to direct the search^29^. BO enhances its surrogate model with newly collected information, making it highly effective for computationally intensive machine learning applications and outperforming alternative methods in complex optimization scenarios. There are very few reports available on the detailed analysis of ML techniques with HPO in ISFET-based bio/chemical sensing. The stacking ensemble learning (SEL) method is further used to boost prediction accuracy that integrate multiple base models^30^. Numerous researchers highlight the contributions of Dasarathy et al. as foundational to ensemble learning^31^. Ensemble learning techniques teach many base learners and integrate their predictions to get enhanced performance. We have proposed HPO and SEL frameworks in XGBoost algorithm that can be effective in pH prediction for the unintended variations caused by the thickness of dielectric, doping concentration, and temperature in ISFET.

In this work, a numerical model of Al_2_O_3_-gated ISFET was developed to generate the I_DS_–V_DS_ characteristics for training the ML technique. The ISFET-based pH sensor was simulated by varying thickness of dielectric (T_OX_), n-type doping of source and drain region (N_D_), and temperature (T). The XGBoost regressor was used to estimate the pH level from ISFET. Further, the hyperparameters of the XGBoost model were optimized using RS, GS and BO techniques. HPO with superior performance metrics was then used for the prediction of pH level. To improve the accuracy of predictions, XGBoost was stacked with multiple learners to train an optimum ML framework. Finally, the key performance metrics, such as coefficient of determination (R^2^), mean absolute error (MAE) and mean squared error (MSE), were obtained from the optimized and stacked XGBoost model for evaluation.

## FEM modelling of ISFET

### Design of ISFET-based pH sensor

**Fig. 1:**
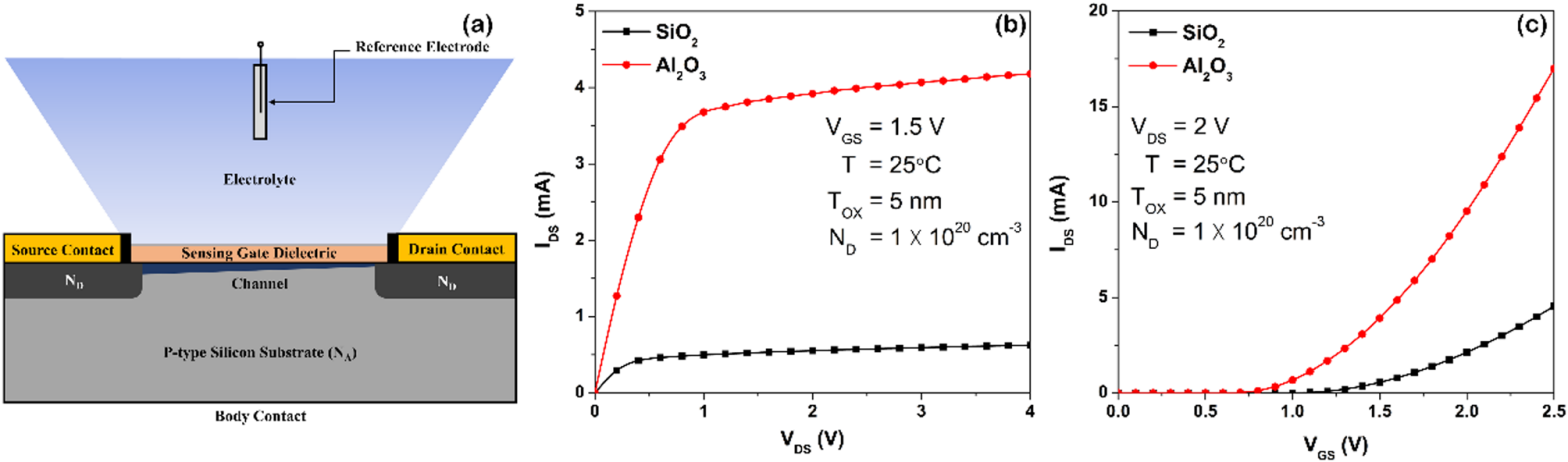
(**a**) Schematic representation of ISFET-based pH sensor, (**b**) Output and (**b**) Transfer characteristics of Al_2_O_3_ and SiO_2_-gated ISFET-based pH sensors at pH = 7.

A 2-dimensional (2D) numerical model of ISFET-based pH sensor was developed considering surface electrochemical reactions and charge density. The modelling study of the proposed Al_2_O_3_-gated ISFET-based pH sensor was carried out with the help of the finite element method (FEM)-based COMSOL Multiphysics tool. The essential parameters including geometry, meshing profile, material properties, suitable physics along with associated boundary conditions were supplied. The modules such as mathematics, semiconductor, transport of diluted species, and electrostatics were used to carry out the simulations of ISFET. The physical model of ISFET device was developed using Poisson-Boltzmann statistics, site-binding theory and Fermi-Dirac carrier model^32,33^. COMSOL multiphysics was used to simulate these equations, focusing on the distribution of electrostatic potential, surface charge density, and electron-hole carrier density. The important equations needed for developing the ISFET model are provided as Eq. (S1) to Eq. (S5) in the supplementary material. The schematic representation of ISFET-based pH sensor is illustrated in Fig. 1a. The geometrical parameters utilized in a simulation study of ISFET are listed in Table 1. The entire structure of ISFET was modelled on 5 nm oxide on the silicon substrate, having width of 2 μm and length of 600 nm. The doping profiles of ISFET were modelled with analytical doping to locate p-type silicon substrate and heavily-doped n-type (n^+^) source-drain domains. The length of source and drain contacts were assumed to be identical. The supporting electrolyte was modelled using the transport of diluted species with its relative permittivity (Ɛ_el _= 78.5) and 0.1 M concentration. The external reference electrode (Ag/AgCl) was illustrated as a bulk electrolyte potential boundary. In addition, the gate dielectric materials, such as Al_2_O_3_ and SiO_2_, were evaluated to study the ISFET-based pH sensing phenomenon. The electrochemical properties of Al_2_O_3_ and SiO_2_ used in modelling are mentioned in Table 2.

**Table 1:** Design parameters of ISFET-based pH sensor.

Parameter	Symbol	Value
Width of ISFET	W	2 µm
Length of ISFET	L	600 nm
Thickness of dielectric	T_OX_	5 nm
Length of contacts	t_C_	100 nm
Doping of p-type silicon substrate	N_A_	1 × 10^17^ cm^−3^
Doping of n-type source and drain	N_D_	1 × 10^20^ cm^−3^
Relative permittivity of SiO_2_	Ɛ_SiO2_	3.9
Relative permittivity of Al_2_O_3_	Ɛ_Al2O3_	14
Temperature	T	25 °C

**Table 2:** Electrochemical properties of Al_2_O_3_ and SiO_2_ as gate dielectric materials.

Parameter and unit	Symbol	Al_2_O_3_	SiO_2_
Positive surface dissociation constant (M)	K_a_	10^–10^	10^–6^
Negative surface dissociation constant (M)	K_b_	10^–6^	100
No. of available surface sites per unit area (cm^−2^)	N_Sil_	8 × 10^14^	5 × 10^14^

### Analysis of Al_2_O_3_-gated ISFET model

Firstly, the performance of the developed ISFET model was assessed for Al_2_O_3_ and SiO_2_ as gate dielectric materials with I–V characteristics keeping pH level of 7. The output (I_DS_–V_DS_) and transfer (I_DS_–V_GS_) characteristics were obtained utilizing Al_2_O_3_ and SiO_2_-gated ISFET models, as illustrated in Fig. 1b and c, respectively. The Al_2_O_3_-gated ISFET model exhibits more desirable characteristics with greater I_DS_ magnitude and lower V_TH_ as compared to SiO_2_. The enhanced response of Al_2_O_3_-gated ISFET was observed due to improved oxide capacitance and high number of available surface sites. Consequently, Al_2_O_3_ was used as a gate dielectric material in ISFET-based pH sensor model for further analysis.

**Fig. 2:**
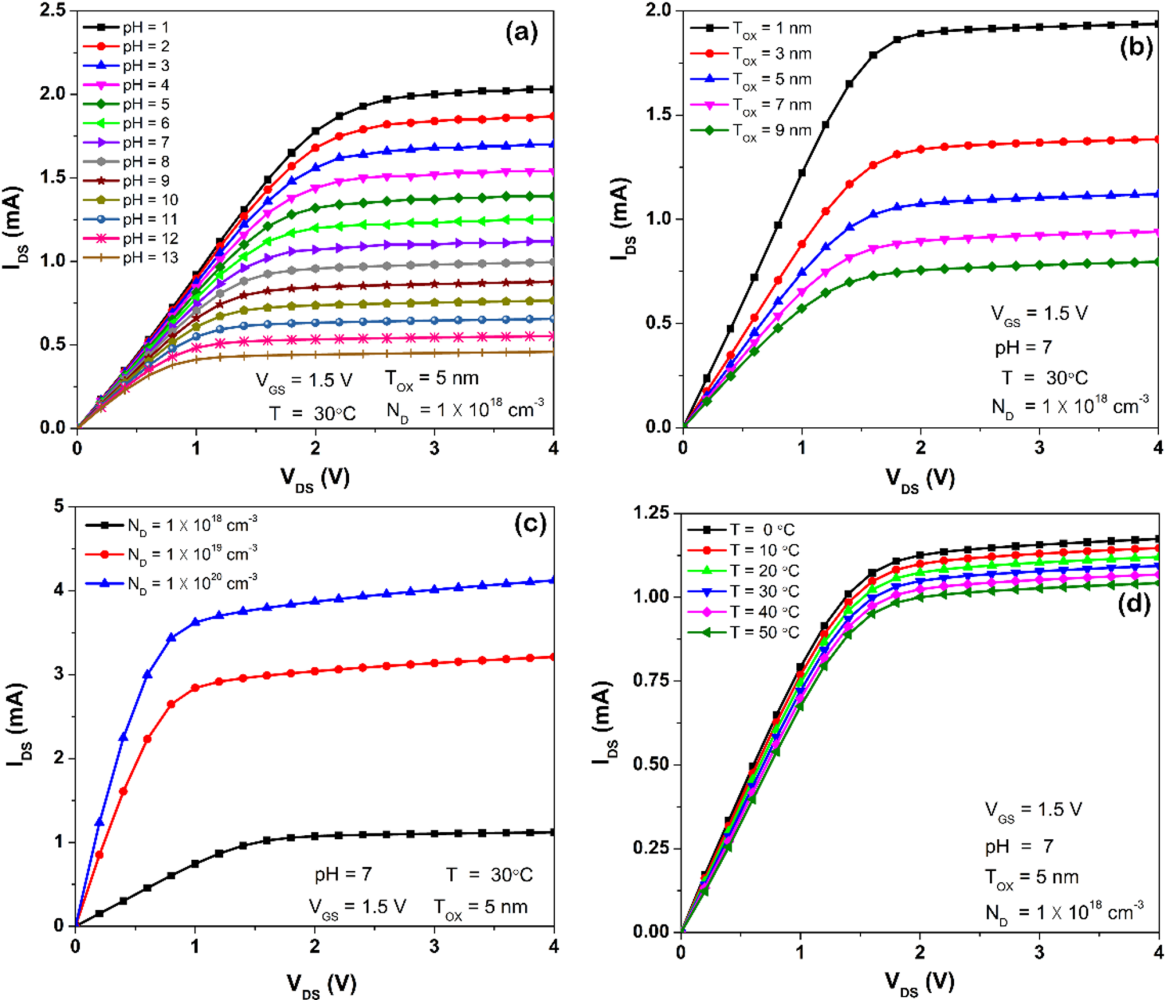
Output characteristics of Al_2_O_3_-gated ISFET due to influence of different parameters such as: (**a**) pH level (1 to 13), (**b**) Thickness of Al_2_O_3_–T_OX_ (1–9 nm), (**c**) n + doping—N_D_ (10^18^ to 10^20^ cm^−3^) and (**d**) Temperature—T (0–50 °C).

The I_DS_–V_DS_ characteristics were obtained to evaluate the Al_2_O_3_-gated ISFET model for variations in operating conditions while sensing, as depicted in Fig. 2. It encompasses variations in pH level, T_OX_, N_D_, and T. The pH level of the electrolyte was varied from 1 to 13, and T_OX_ was increased from 1 to 9 nm. Additionally, N_D_ was varied from 10^18^ cm^−3^ to 10^20^ cm^−3^, while the temperature range taken into account was from 0 to 50 °C. Figure 2a illustrates that as an increase in the pH level of the electrolyte resulted in a reduction in I_DS_. When T_OX_ is substantially decreased, the magnitude of the I_DS_ increases, as seen from Fig. 2b. In this scenario, a reduction in T_OX_ led to a corresponding change in the threshold voltage, which resulted in an exponential increase in I_DS_. The I_DS_–V_DS_ characteristics in Fig. 2c indicates an upward shift with higher N_D_, since electrons in both source and drain regions increased substantially. This phenomenon injected more electrons into the channel underneath the insulator, enhancing current flow through it. Moreover, a rise in temperature around ISFET led to a downward shift of the I_DS_–V_DS_ characteristics, hence decreasing I_DS_, as shown in Fig. 2d. Thus, the geometrical and electrochemical parameters with temperature dependence make ISFET susceptible to variations in mobility, carrier concentration, threshold voltage, and drain-to-source current.

### Generation of dataset for ML techniques

**Fig. 3:**
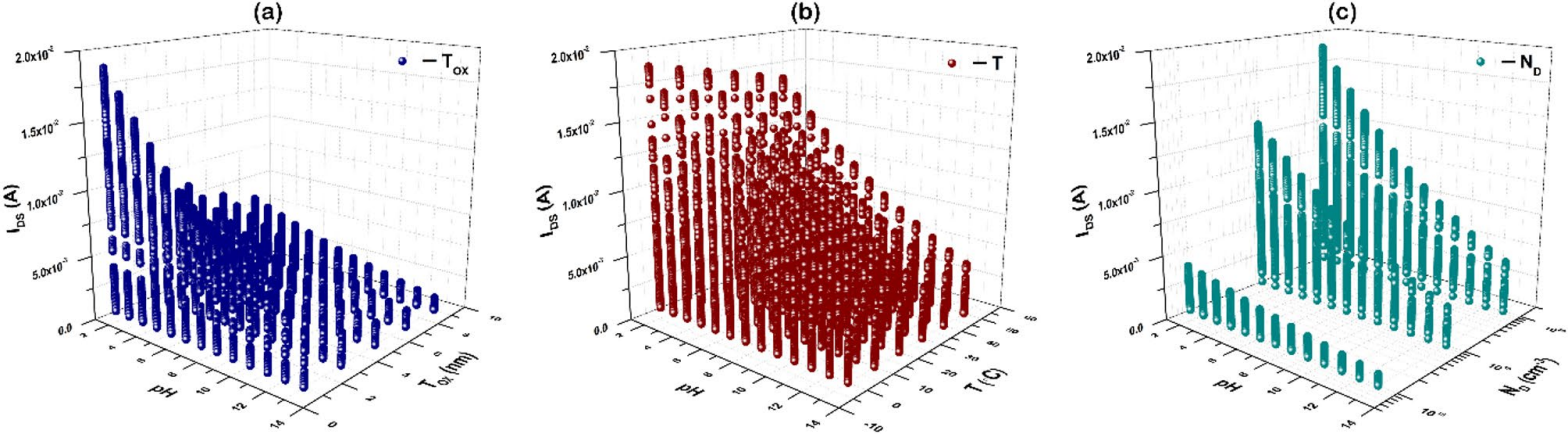
Representation of data generated from Al_2_O_3_-based ISFET-based pH sensor for variations in (**a**) Thickness of Al_2_O_3_ (T_OX_), (**b**) Temperature (T) and (**c**) Doping concentration (N_D_).

After the assessment of ISFET model, I_DS_-V_DS_ characteristics were used to generate a dataset to train, test and validate the ML models. This dataset consists of 24,575 datapoints of I_DS_–pH response with various features such as T_OX_, N_D_, and T, as represented in Fig. 3. The associated pH level was considered as the output feature to be predicted. The change in I_DS_ response as a result of change in Al_2_O_3_ thickness, temperature around ISFET, and n^+^ doping of source/drain region are evident in Fig. 3a, b and c, respectively. The sample snapshots of the labelled dataset are provided in Fig. S1 of the supplementary material.

## Methodology

In this section, the methodology for implementation of XGBoost framework was discussed in detail. This study establishes a framework for effectively predicting the pH level of Al_2_O_3_-gated ISFET. By using stacking ensemble learning (SEL), the prediction accuracy of pH was enhanced with XGBoost regressor as a meta learner. The hyperparameter tuning becomes particularly important in this work for high computing efficiency, accurate learning, and outlier reduction. Additionally, SEL is powerful method for integrating best learners via adaptive learning with reduced variance and reliable decision-making. XGBoost is an enhancement of the gradient boosting decision tree algorithm, designed to enhance both the running speed and the accuracy of the model. One of the significant and fundamental works in XGBoost was introduced by Chen and Guestrin, utilizing a scalable tree boosting approach^34^. It incorporates decision trees as the base classifiers, with the complexity of the trees regulated by a modification of the objective function as presented in Eq. 1 and 2^35,36^:

1 $${\text{L}}_{\text{ XGB}}=\sum_{\text{i}=1}^{\text{N}}\text{L }({\text{ y}}_{\text{i}} ,{\text{ f}}_{\text{t}} \left({\text{x}}_{\text{i}}\right) )+\sum_{\text{m}=1}^{\text{M}}\Omega ({\text{ f}}_{\text{t}} )$$

2 $$\Omega \left( {\text{f}}_{\text{t}} \right)=\upgamma {\text{T}}_{\text{r}}+ \frac{1}{2}\Lambda { \Vert \text{w}\Vert }^{2}$$

where L indicates an objective function that represents the error between observed and predicted data, t is the index of iteration for the optimization procedure, ${\text{f}}_{\text{t}}$ is the model of the tth tree, Ω is a regulation term that penalizes the complexity of the model, T_r_ is the number of tree leaves, γ & $\Lambda$ are penalty coefficients. w is a vector containing each leaf’s score. The value of γ controls the minimum loss reduction gain needed to split an internal node.

### Framework of XGBoost algorithm

**Fig. 4:**
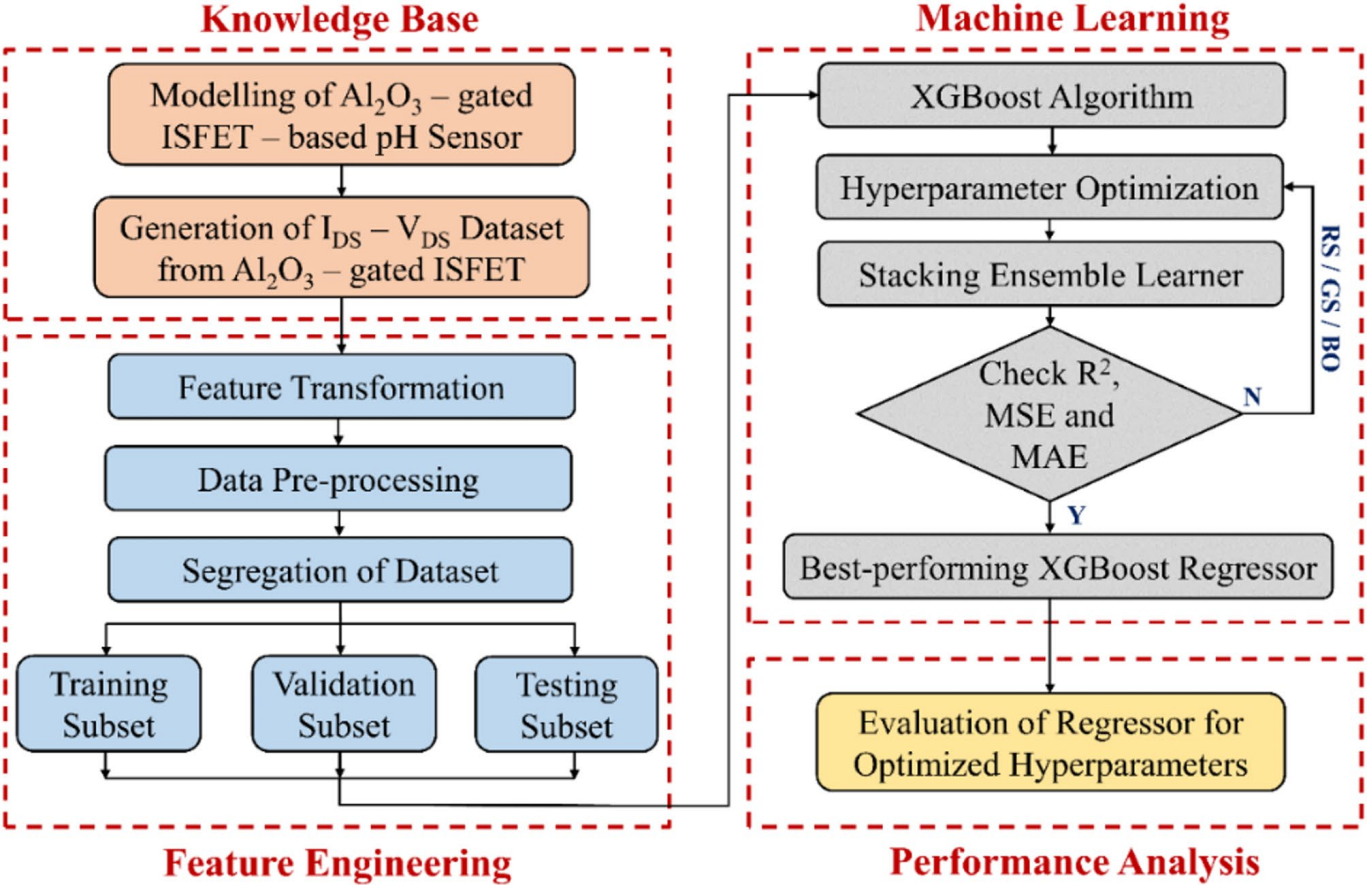
Basic schematic representation of XGBoost framework.

The workflow of the ML technique comprises several steps involving data acquisition to construct input for subsequent learning and building the XGBoost model. This is succeeded by the identification, validation, and performance assessment of the model. This workflow is essential for accurately predicting features from the provided dataset. The process of the proposed XGBoost framework is illustrated in Fig. 4. It is categorized into four distinct stages, i.e., knowledge base, feature engineering, machine learning, and performance analysis stage. The I_DS_–V_DS_ dataset generated from Al_2_O_3_-gated ISFET model was primarily utilized in the knowledge base, including various input and output features. Furthermore, the feature engineering stage consists of transformation, pre-processing and data segregation steps. The second-order polynomial feature transformation was utilized to calculate the polynomial combinations of existing features. These combinations were subsequently applied to generate additional features for identifying a non-linear correlation among the features. The pre-processing procedures were carried out to normalize the values from the generated dataset to a comparable scale. The dataset was further divided randomly into training and validation subsets comprising 75% of the whole dataset, with the remaining 25% allocated for testing subset. The training subset was used to extract valuable insights from the provided I-V characteristics to train the ML model. While the validation subset was applied to determine and tune the hyperparameters. The testing subset was specifically utilized to evaluate the algorithmic performance.

During the machine learning stage, the XGBoost regression model was trained to learn the correlation between the input and output features. The search spaces for XGBoost hyperparameters were also defined using appropriate data types, attributes, and ranges based on prior knowledge and literature. This work utilized several well-known hyperparameter optimization (HPO) techniques and evaluated them to attain minimal error along with maximum accuracy in prediction. This procedure continued iteratively until the XGBoost algorithm yielded superior results in regression. The best-performing HPO technique was then used for the stacking ensemble learning (SEL) approach. The outcomes of the standalone XGBoost regression model were stacked (base and final estimator) to train a system aimed at enhancing prediction accuracy. Finally, an in-depth evaluation of the XGBoost algorithm was carried out using significant performance metrics such as R^2^, MSE and MAE. The R^2^ score is crucial for assessing algorithm performance in relation to variance, interpretability, and overfitting. MSE imposes a more severe penalty on outliers, making it sensitive to large deviations. Whereas MAE exhibits more robustness to outliers and noise.

## Performance evaluation of machine learning techniques

### Study of XGBoost hyperparameters

The hyperparameters of any ML technique are the configuration variables utilized to regulate the learning process and adjusted to enhance performance^26^. The hyperparameters of the XGBoost algorithm are often divided into two key categories: tree-specific and learning task-specific. Some of the crucial tree-specific hyperparameters encompass maximum depth, minimum child weight, and n estimators. The learning task-specific hyperparameters, such as the learning rate (η), α, and λ parameter, govern the overall behaviour of the model. One of the tree-specific hyperparameters, the maximum depth of each tree, is utilized to capture more intricate information in the deeper trees. The minimum child weight refers to a minimum sum of instance weight needed in a child node of a decision tree^37^. The n-estimators parameter denotes the upper limit on the number of gradient-boosted trees employed in building a model. Moreover, the learning rate (η) represents a step size at each iteration while optimizing the objective function. The parameters α and λ predominantly affect the regularization terms applied to the weights. The value of α imposes a penalty on the model for possessing non-zero coefficients. Whereas λ helps mitigate overfitting by penalizing significant coefficients, encouraging a simpler and more generalizable model.

**Fig. 5:**
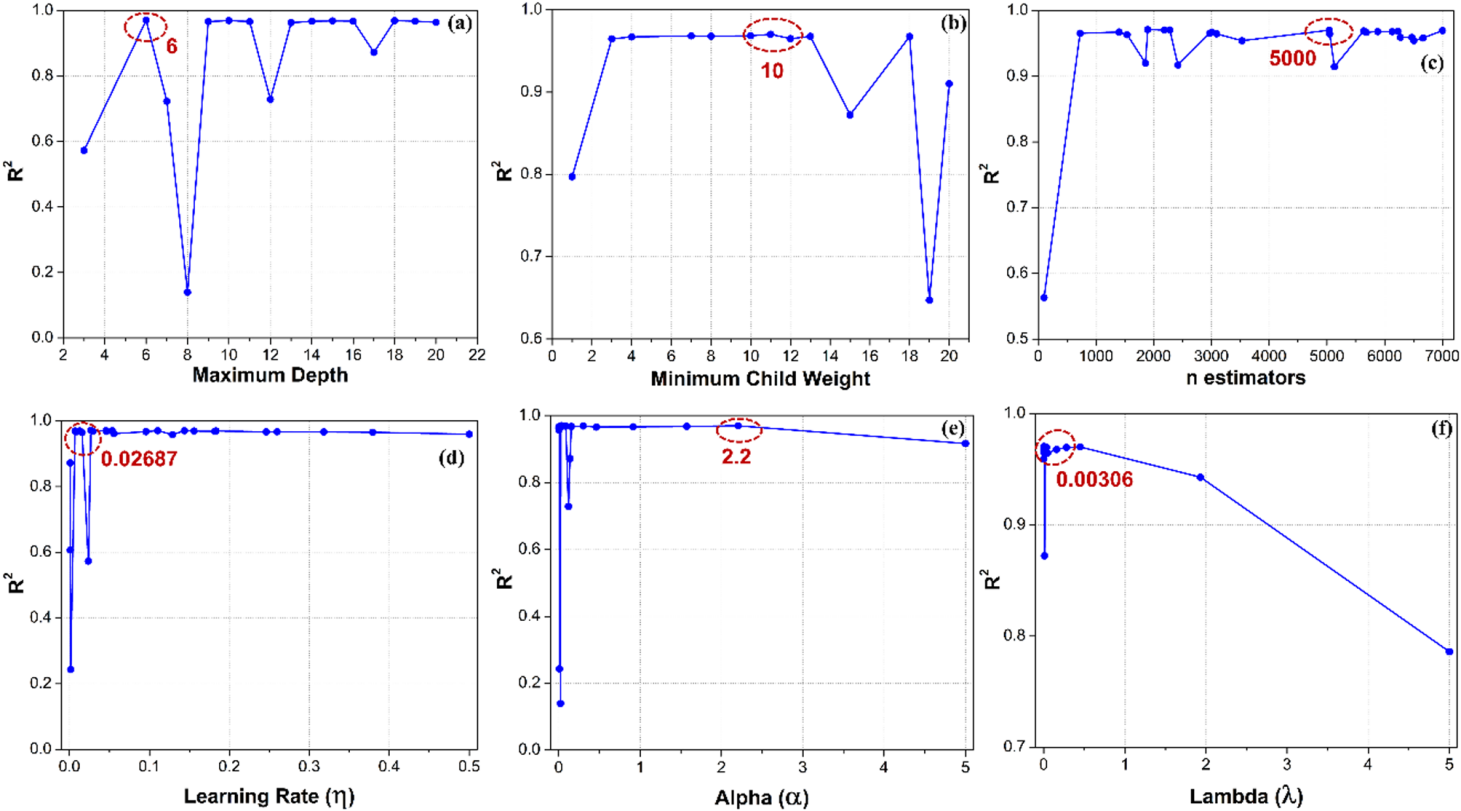
R^2^ obtained for various key XGBoost hyperparameters: (**a**) Maximum depth, (**b**) Minimum child weight, (**c**) n estimators, (**d**) Learning rate (η), (**e**) α and (**f**) λ.

Every ML technique possesses a unique set of hyperparameters that must be adjusted to get the best results for each dataset^24^. We have independently evaluated the effect of each hyperparameter in XGBoost regressor to ensure accurate predictions before systematic tuning. The study was conducted by examining the local neighbourhood of the specified hyperparameter and assessing the performance variations with the help of R^2^ score^25^. We ensured that all hyperparameters are within their appropriate range and datatype, since they are highly sensitive to minor modifications and directly influence model performance^38^. We also checked that overfitting is mitigated by employing cross-validation, regularization (utilizing α and λ), and early stopping. To determine the optimal range of hyperparameters, R^2^ values were calculated for different key hyperparameters used in XGBoost as shown in Fig. 5. The R^2^ values of key hyperparameters, such as maximum depth, minimum child weight, n estimators, η, α, and λ, is illustrated in Fig. 5a–f, respectively. The encircled regions shown in Fig. 5 denote the probable optimal ranges or search spaces. The optimal search space helps in optimizing the hyperparameter, preventing overfitting. This also helps to build a reliable HPO strategy which can improve optimization efficiency by avoiding redundant search spaces of hyperparameters.

### Hyperparameter optimization (HPO) in XGBoost regression

Generally, the hyperparameter optimization (HPO) technique aims to improve model reproducibility, minimize human involvement, and boost the performance of ML techniques. The systematic methodology for HPO encompasses techniques such as random search (RS), grid search (GS), and Bayesian optimization (BO). The efficacy of HPO techniques was evaluated using validation subset and a suitable approach was then utilized. The R^2^ score, MSE and MAE for RS, GS and BO techniques are illustrated in Table 3. It is evident that BO exhibits optimized R^2^ score of 0.9795, MSE of 0.048 and MAE of 0.21. BO has shown comparable or superior accuracy while requiring significantly fewer number of evaluations. It also outperformed GS and RS due to the use of probabilistic model. This model requires less computational resources and efficiently navigates the hyperparameter space that maximize output prediction accuracy. It also focuses on the most promising areas of the search space, making it faster.

**Table 3:** Hyperparameter optimization techniques used in XGBoost regressor.

Sr. No	HPO technique	R^2^	MSE	MAE
1	Random search (RS)	0.9554	0.112	0.32
2	Grid search (GS)	0.9658	0.062	0.26
3	Bayesian optimization (BO)	0.9795	0.048	0.21

**Fig. 6:**
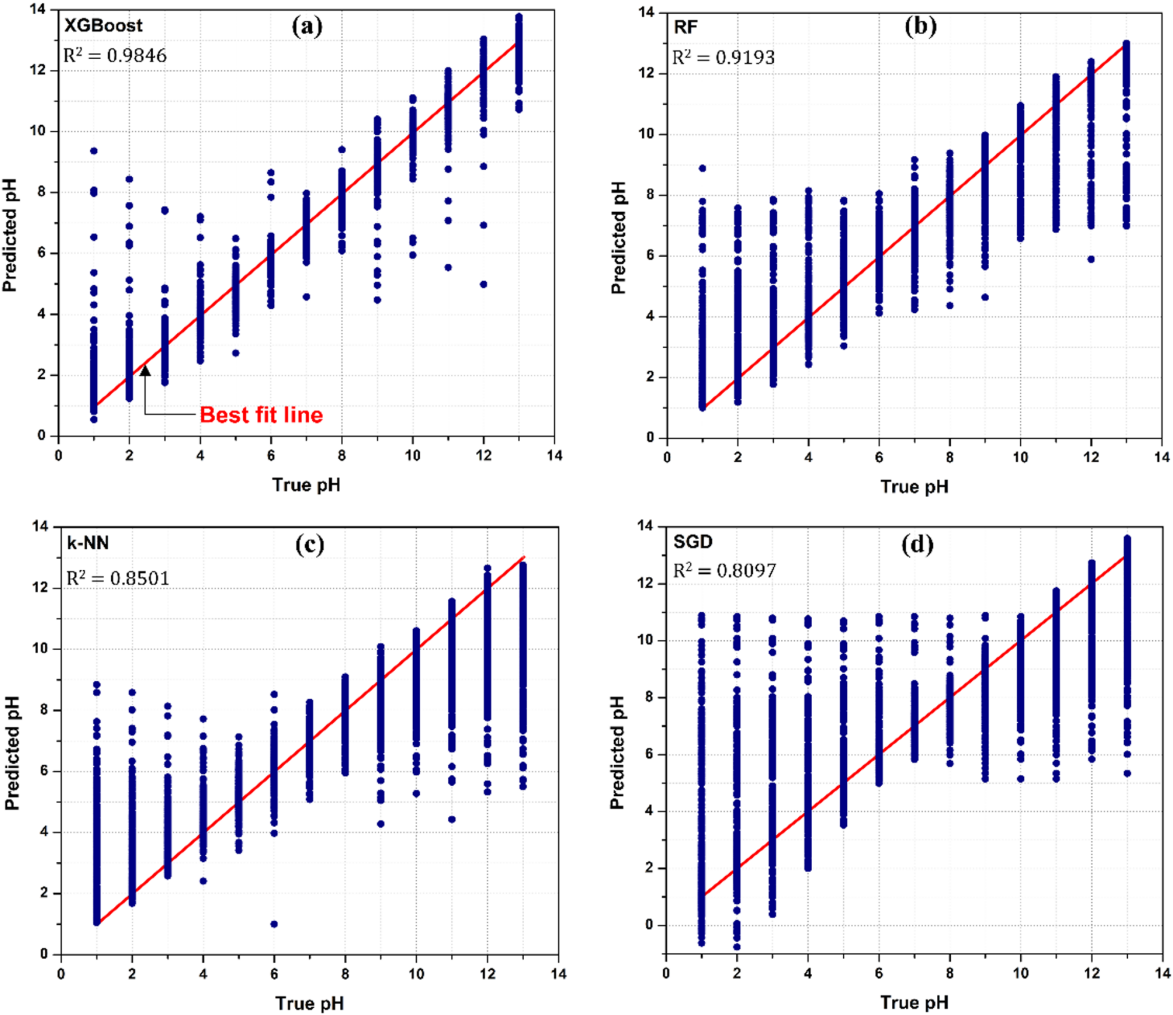
Scatter plots of (**a**) XGBoost, (**b**) RF, (**c**) k-NN and (**d**) SGD regressors with associated R^2^ scores.

Upon evaluating BO as a better-performing HPO algorithm, the model eventually provided the following hyperparameters with their respective search ranges and optimal values, as depicted in Table 4. The BO-based XGBoost regressor exhibited enhanced performance with the hyperparameters such as maximum depth of 6 and a minimum child node weight of 10. The optimal values of n estimators and learning rate should be set to 5000 and 0.02687, respectively, for accurate prediction of pH level. The higher R^2^ score were accomplished for α = 2.2 and λ = 0.00306. The optimal hyperparameter values obtained using BO-based HPO technique are identical to the maximum R^2^ value presented in Fig. 6.

**Table 4:** Significant hyperparameters of XGBoost algorithm with their ranges and optimal values based on Bayesian Optimization (BO) technique.

Hyperparameter	Format type	Search range	Optimal value
Maximum depth	Integer	[3, 20]	6
Minimum child weight	Integer	[1, 20]	10
n estimators	Integer	[100, 7000]	5000
Learning rate (η)	Real	[0.001, 0.5]	0.02687
α	Real	[0.001, 5.0]	2.2
λ	Real	[0.001, 5.0]	0.00306

### Stacking ensemble learning (SEL) approach for XGBoost regression

Most of the standalone ML models are unlikely to entirely capture the underlying structure of the data necessary for optimal predictions. Research indicates that ML algorithms may occasionally exhibit high variance and low precision, resulting in suboptimal performance^39^. In order to enhance the level of prediction accuracy further, the standalone XGBoost regression model was stacked to train an optimal learning system focused on minimizing generalization errors, known as stacking ensemble learning (SEL) method^40^. Successive-level models often utilize information from the lower-level models, with each model delivering its most precise estimate. The multiple models are generally trained in SEL on the same training dataset and their predictions are used as input for a second-level model known as meta-learner. The SEL approach combines diverse models to make the most of their strengths and demonstrates improved generalization capability over the individual learners. The SEL, along with the BO-based XGBoost regressor, was trained here and assessed using identical training and testing datasets.

SEL method was used to achieve higher accuracy, especially when standalone models underperform. SEL integrates several base models with a meta-learner to capitalize on their complementary strengths while addressing nonlinear interactions and data heterogeneity. In this study, linear regression (LR) and random forest (RF) were used as the base models, while the optimized XGBoost model served as the meta-learner. An LR was chosen for its interpretability and computational efficiency, serving as a baseline and providing insights into the relationships between features for pH prediction. To enhance the prediction further, an RF regressor was used to capture potential non-linearities within the ISFET data, while maintaining relative computational tractability. This layered approach facilitates a progressive assessment of model complexity and effectiveness within the constraints of our generated dataset. Table 5 illustrates the performance analysis of the XGBoost regression with and without HPO and SEL using R^2^, MSE and MAE. It is clearly demonstrated in Table 5 that the upgraded XGBoost regressor yielded the greatest R^2^ score (0.9846) and lowest MSE and MAE values (0.2342 and 0.3862), compared to the standalone XGBoost models with and without HPO. Therefore, the pH level of Al_2_O_3_-gated ISFET was efficiently predicted by the upgraded XGBoost model to account for unintended variations caused by T_OX_, N_D_, and T.

**Table 5:** Performance analysis of XGBoost regressors.

Sr. No	XGBoost regressor models	R^2^	MSE	MAE
1	Standalone models without HPO	0.9559	0.4026	0.5064
2	Standalone model with HPO	0.9795	0.2870	0.4275
3	Upgraded model (BO and SEL)	0.9846	0.2342	0.2317

### Comparison of upgraded XGBoost regressor with other ML algorithms

The performance of upgraded (BO and SEL-based) XGBoost regressor was evaluated against other conventional ML techniques, including random forest (RF), k-nearest neighbour (k-NN), and stochastic gradient descent (SGD). The hyperparameters of these conventional models were optimized and compared against upgraded XGBoost regressor. The scatter plots of these ML techniques are illustrated in Fig. 6. The red line in Fig. 6 denotes an accurate line of prediction, whereas the blue dots signify the predicted value of pH level (ranging from 1 to 13). It is observed from Fig. 6a, b and c that the RF and k-NN regressors, which appear to have worked moderately, however produced a lower R^2^ score than XGBoost algorithm. Consequently, SGD is the least effective model for the given dataset, yielding lowest R^2^, as depicted in Fig. 6d. Moreover, Table 6 summarizes the comprehensive outcomes of different ML regressors by computing appropriate performance metrics, such as R^2^ score, MAE, and MSE, using identical test dataset. The algorithms excluding XGBoost regression reported significant values of errors. It was observed that MSE and MAE values of RF, k-NN and SGD are approximately two to seven times higher than those of the upgraded XGBoost regressors. The upgraded XGBoost regression model provided the highest R^2^ score, lowest MAE and MSE values. It signifies a more accurate prediction of pH level by minimizing generalization errors. In addition, assessing Tables 5 and 6 reveals that R^2^ score of the standalone XGBoost model without hyperparameter optimization (0.9559) surpasses that of the RF, k-NN, and SGD regressors. All these findings indicate that the upgraded (BO and SEL-based) XGBoost regressor outperformed the RF, k-NN, and SGD regressors, achieving R^2^ = 0.9846, MSE = 0.2342, and MAE = 0.2317.

**Table 6:** Performance evaluation of different ML regressors.

Sr. No.	ML regressor	R^2^	MSE	MAE
1	XGBoost	0.9846	0.2342	0.2317
2	RF	0.9193	1.1411	0.4958
3	k-NN	0.8501	1.1201	0.9264
4	SGD	0.8097	1.6907	0.9152

**Fig. 7:**
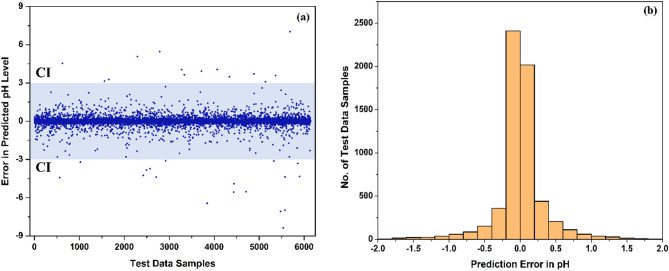
(**a**) Prediction Error with confidence internal (CI) and (**b**) Error Distribution in test results of upgraded (BO and SEL) XGBoost regressor.

Additionally, the statistical analysis was carried out for the predicted pH levels obtained from the test samples of the upgraded XGBoost regressor, which included 25% of the actual dataset (totalling 6143 samples). The errors in the prediction of pH levels and error distribution of the upgraded XGBoost regressor are presented in Fig. 7a and b, respectively. It is observed from Fig. 7a that the majority of test data samples have prediction errors nearly equal to zero. In addition, 75.64% of test samples (4647 out of 6143) exhibit a prediction error in pH of ± 0.2, as illustrated in Fig. 7b. The results of statistical error analysis validate the accuracy and precision of the upgraded XGBoost regressor in predicting pH levels.

## Conclusion

This work offers a comprehensive analysis of the XGBoost regressor, incorporating suitable hyperparameter optimization (HPO) and stacking ensemble learner (SEL) to predict pH levels of ISFET. A numerical model of Al_2_O_3_-gated ISFET-based pH sensor was developed. The performance of ISFET was assessed by varying T_OX_, N_D_, and T. The I_DS_–V_DS_ characteristics were obtained from ISFET. The XGBoost algorithm was employed utilizing I_DS_–V_DS_ dataset to train the regression models. The performance of each hyperparameter of XGBoost algorithm was analyzed to ensure the probable optimal range of operation. The HPO techniques such as RS, GS and BO were used to optimize the hyperparameters. Among these HPO, BO outperformed giving R^2^ of 0.9795, MSE of 0.048 and MAE of 0.21. The optimized XGBoost regressor was further integrated with the stacking ensemble learner (SEL) to improve the accuracy of prediction. The performance of the upgraded (BO and SEL-based) XGBoost regressor was compared with that of optimized RF, k-NN, and SGD algorithms. The upgraded XGBoost regressor resulted in R^2^ of 0.9846, MSE of 0.2342 and MAE of 0.2317. In addition, the statistical error analysis was conducted for the prediction by the XGBoost regressor and it revealed that 75.64% of test samples lay within ± 0.2 prediction error in pH. This study highlights the benefits of XGBoost and the significance of HPO and SEL for accurate prediction of the pH level from Al_2_O_3_-gated ISFET. Hence, the XGBoost regressors based on hyperparameter optimization and stacking ensemble learners are found to be very effective in predicting the pH levels of ISFETs under various operating conditions.

## Electronic supplementary material

Below is the link to the electronic supplementary material.

Supplementary Material 1

## Data Availability

The datasets generated and/or analyzed during the current study are available from the corresponding author upon reasonable request.

## Code availability

This information is available from the corresponding author upon reasonable request.
