# Supplementary material for: Enhancing pH prediction accuracy in Al2O3 gated ISFET using XGBoost regressor and stacking ensemble learning
Source: Sci Rep. 2025 Jun 1;15:19197. doi: 10.1038/s41598-025-04530-2 (PMC12127451; doi:10.1038/s41598-025-04530-2)
Supplement: Supplementary file 1 — Supplementary Material 1 [file 41598_2025_4530_MOESM1_ESM.pdf]

# Supplementary Material

## Enhancing pH Prediction Accuracy in $Al_2O_3$ gated ISFET using XGBoost Regressor and Stacking Ensemble Learning

Ashirbad Panda[1], Rishikesh Datar[1], Shreyas Deshpande[1], and Gautam Bacher[1,*]
[1]Department of Electrical and Electronics Engineering, BITS Pilani K K Birla Goa Campus, Goa- 403726, India.
[*]Corresponding author. E-mail: ggb@goa.bits-pilani.ac.in.

- **Important equations used for $Al_2O_3$-gated ISFET-model:**

The important equations, which were used to develop ISFET model using Poisson-Boltzmann statistics, Fermi-Dirac carrier model and site-binding theory, are mentioned below:

1) Poisson equation:

$$-\nabla \cdot \left(\frac{\varepsilon_0 \varepsilon_r}{q} \nabla V\right) = -n + p + N_D^+ - N_A^- \qquad \text{Eq. (S1)}$$

Where,

$\varepsilon_0$ = Vacuum permittivity

$\varepsilon_r$ = Relative permittivity of the material

q = Elementary charge

V = Electric potential

n = electron concentration

p = hole concentration

$N_D$ = Donor doping level

$N_A$ = Acceptor doping level

2) Electron/hole continuity equation:

$$\nabla \cdot J_n = qU_n \qquad \text{Eq. (S2)}$$

$$\nabla \cdot J_p = qU_p \qquad \text{Eq. (S3)}$$

Where,

$J_n$ = Current density of electrons

$J_p$ = Current density of holes

$U_n$ = Net generation-recombination rate for electrons.

$U_p$ = Net generation-recombination rate for holes.

3) Conduction band edge potential:

$$E_c = -(V + \chi_0 + E_{g0}) \qquad \text{Eq. (S4)}$$

Where,

$E_c$ = Conduction band edge energy

V = Electrostatic potential

$\chi_0$ = Electron affinity of the semiconductor

$E_{g0}$ = Bandgap energy at 0 K

4) Surface charge density using site-binding model:

$$\sigma_0 = -qN_s\left(\frac{[H^+]^2 - K_a K_b}{K_a K_b + K_a[H^+] + [H^+]^2}\right) \quad \text{Eq. (S5)}$$

Where,

q = Elementary charge

$N_s$ = Density of surface sites (sites per $m^2$)

$K_a$ and $K_b$ = Equilibrium constants for protonation/deprotonation

$[H^+]$ = Hydrogen ion concentration i.e., $10^{-pH}$

- **Labelled Dataset for ML algorithms:**

The sample snapshots of the labelled dataset are provided below:

| pH | tox (nm) | T0 (degC) | Nd (1/cm^3) | VGS (V) | VDS (V) | IDS (A) |
|---|---|---|---|---|---|---|
| 1 | 1 | 0 | 1.00E+18 | 1.5 | 0 | -1.70E-08 |
| 1 | 1 | 0 | 1.00E+18 | 1.5 | 0.2 | 2.13E-04 |
| 1 | 1 | 0 | 1.00E+18 | 1.5 | 0.4 | 4.43E-04 |
| 1 | 1 | 0 | 1.00E+18 | 1.5 | 0.6 | 6.99E-04 |
| 1 | 1 | 0 | 1.00E+18 | 1.5 | 0.8 | 9.76E-04 |
| 1 | 1 | 0 | 1.00E+18 | 1.5 | 1 | 0.00127178 |
| 1 | 1 | 0 | 1.00E+18 | 1.5 | 1.2 | 0.001583189 |
| 1 | 1 | 0 | 1.00E+18 | 1.5 | 1.4 | 0.001901408 |
| 1 | 1 | 0 | 1.00E+18 | 1.5 | 1.6 | 0.002213622 |
| 1 | 1 | 0 | 1.00E+18 | 1.5 | 1.8 | 0.002502155 |
| 1 | 1 | 0 | 1.00E+18 | 1.5 | 2 | 0.002744721 |
| 1 | 1 | 0 | 1.00E+18 | 1.5 | 2.2 | 0.002919984 |
| 1 | 1 | 0 | 1.00E+18 | 1.5 | 2.4 | 0.0030212 |
| 1 | 1 | 0 | 1.00E+18 | 1.5 | 2.6 | 0.003066383 |
| 1 | 1 | 0 | 1.00E+18 | 1.5 | 2.8 | 0.003084363 |
| 1 | 1 | 0 | 1.00E+18 | 1.5 | 3 | 0.003093258 |
| 1 | 1 | 0 | 1.00E+18 | 1.5 | 3.2 | 0.003099378 |
| 1 | 1 | 0 | 1.00E+18 | 1.5 | 3.4 | 0.003104476 |
| 1 | 1 | 0 | 1.00E+18 | 1.5 | 3.6 | 0.003109061 |
| 1 | 1 | 0 | 1.00E+18 | 1.5 | 3.8 | 0.00311332 |
| 1 | 1 | 0 | 1.00E+18 | 1.5 | 4 | 0.003117327 |
| 1 | 1 | 0 | 1.00E+19 | 1.5 | 0 | -1.04E-07 |
| 1 | 1 | 0 | 1.00E+19 | 1.5 | 0.2 | 0.001418511 |
| 1 | 1 | 0 | 1.00E+19 | 1.5 | 0.4 | 0.003032303 |
| 1 | 1 | 0 | 1.00E+19 | 1.5 | 0.6 | 0.004865629 |

| pH | tox (nm) | T0 (degC) | Nd (1/cm^3) | VGS (V) | VDS (V) | IDS (A) |
|---|---|---|---|---|---|---|
| 1 | 3 | 50 | 1.00E+18 | 1.5 | 0 | -3.99E-09 |
| 1 | 3 | 50 | 1.00E+18 | 1.5 | 0.2 | 2.18E-04 |
| 1 | 3 | 50 | 1.00E+18 | 1.5 | 0.4 | 4.30E-04 |
| 1 | 3 | 50 | 1.00E+18 | 1.5 | 0.6 | 6.51E-04 |
| 1 | 3 | 50 | 1.00E+18 | 1.5 | 0.8 | 8.79E-04 |
| 1 | 3 | 50 | 1.00E+18 | 1.5 | 1 | 0.00111481 |
| 1 | 3 | 50 | 1.00E+18 | 1.5 | 1.2 | 0.001355337 |
| 1 | 3 | 50 | 1.00E+18 | 1.5 | 1.4 | 0.001595369 |
| 1 | 3 | 50 | 1.00E+18 | 1.5 | 1.6 | 0.001827082 |
| 1 | 3 | 50 | 1.00E+18 | 1.5 | 1.8 | 0.002040569 |
| 1 | 3 | 50 | 1.00E+18 | 1.5 | 2 | 0.00222481 |
| 1 | 3 | 50 | 1.00E+18 | 1.5 | 2.2 | 0.002369409 |
| 1 | 3 | 50 | 1.00E+18 | 1.5 | 2.4 | 0.002468659 |
| 1 | 3 | 50 | 1.00E+18 | 1.5 | 2.6 | 0.002526691 |
| 1 | 3 | 50 | 1.00E+18 | 1.5 | 2.8 | 0.002556954 |
| 1 | 3 | 50 | 1.00E+18 | 1.5 | 3 | 0.002573265 |
| 1 | 3 | 50 | 1.00E+18 | 1.5 | 3.2 | 0.002583636 |
| 1 | 3 | 50 | 1.00E+18 | 1.5 | 3.4 | 0.002591457 |
| 1 | 3 | 50 | 1.00E+18 | 1.5 | 3.6 | 0.00259804 |
| 1 | 3 | 50 | 1.00E+18 | 1.5 | 3.8 | 0.002603919 |
| 1 | 3 | 50 | 1.00E+18 | 1.5 | 4 | 0.002609335 |
| 1 | 3 | 50 | 1.00E+19 | 1.5 | 0 | -2.94E-08 |
| 1 | 3 | 50 | 1.00E+19 | 1.5 | 0.2 | 0.00129902 |
| 1 | 3 | 50 | 1.00E+19 | 1.5 | 0.4 | 0.002616135 |
| 1 | 3 | 50 | 1.00E+19 | 1.5 | 0.6 | 0.003962689 |
| 1 | 3 | 50 | 1.00E+19 | 1.5 | 0.8 | 0.005244124 |
| 1 | 3 | 50 | 1.00E+19 | 1.5 | 1 | 0.006347716 |
| 1 | 3 | 50 | 1.00E+19 | 1.5 | 1.2 | 0.007176144 |

| pH | tox (nm) | T0 (degC) | Nd (1/cm^3) | VGS (V) | VDS (V) | IDS (A) |
|---|---|---|---|---|---|---|
| 9 | 7 | 30 | 1.00E+18 | 1.5 | 0 | -3.17E-10 |
| 9 | 7 | 30 | 1.00E+18 | 1.5 | 0.2 | 1.30E-04 |
| 9 | 7 | 30 | 1.00E+18 | 1.5 | 0.4 | 2.53E-04 |
| 9 | 7 | 30 | 1.00E+18 | 1.5 | 0.6 | 3.73E-04 |
| 9 | 7 | 30 | 1.00E+18 | 1.5 | 0.8 | 4.79E-04 |
| 9 | 7 | 30 | 1.00E+18 | 1.5 | 1 | 5.66E-04 |
| 9 | 7 | 30 | 1.00E+18 | 1.5 | 1.2 | 6.27E-04 |
| 9 | 7 | 30 | 1.00E+18 | 1.5 | 1.4 | 6.62E-04 |
| 9 | 7 | 30 | 1.00E+18 | 1.5 | 1.6 | 6.81E-04 |
| 9 | 7 | 30 | 1.00E+18 | 1.5 | 1.8 | 6.90E-04 |
| 9 | 7 | 30 | 1.00E+18 | 1.5 | 2 | 6.96E-04 |
| 9 | 7 | 30 | 1.00E+18 | 1.5 | 2.2 | 7.01E-04 |
| 9 | 7 | 30 | 1.00E+18 | 1.5 | 2.4 | 7.05E-04 |
| 9 | 7 | 30 | 1.00E+18 | 1.5 | 2.6 | 7.09E-04 |
| 9 | 7 | 30 | 1.00E+18 | 1.5 | 2.8 | 7.12E-04 |
| 9 | 7 | 30 | 1.00E+18 | 1.5 | 3 | 7.15E-04 |
| 9 | 7 | 30 | 1.00E+18 | 1.5 | 3.2 | 7.18E-04 |
| 9 | 7 | 30 | 1.00E+18 | 1.5 | 3.4 | 7.21E-04 |
| 9 | 7 | 30 | 1.00E+18 | 1.5 | 3.6 | 7.23E-04 |
| 9 | 7 | 30 | 1.00E+18 | 1.5 | 3.8 | 7.26E-04 |
| 9 | 7 | 30 | 1.00E+18 | 1.5 | 4 | 7.28E-04 |
| 9 | 7 | 30 | 1.00E+19 | 1.5 | 0 | -7.27E-09 |
| 9 | 7 | 30 | 1.00E+19 | 1.5 | 0.2 | 6.52E-04 |
| 9 | 7 | 30 | 1.00E+19 | 1.5 | 0.4 | 0.001165267 |
| 9 | 7 | 30 | 1.00E+19 | 1.5 | 0.6 | 0.001511378 |
| 9 | 7 | 30 | 1.00E+19 | 1.5 | 0.8 | 0.001677161 |
| 9 | 7 | 30 | 1.00E+19 | 1.5 | 1 | 0.001738272 |
| 9 | 7 | 30 | 1.00E+19 | 1.5 | 1.2 | [illegible] |

| pH | tox (nm) | T0 (degC) | Nd (1/cm^3) | VGS (V) | VDS (V) | IDS (A) |
|---|---|---|---|---|---|---|
| 13 | 7 | 0 | 1.00E+18 | 1.5 | 0 | -2.32E-09 |
| 13 | 7 | 0 | 1.00E+18 | 1.5 | 0.2 | 8.63E-05 |
| 13 | 7 | 0 | 1.00E+18 | 1.5 | 0.4 | 1.70E-04 |
| 13 | 7 | 0 | 1.00E+18 | 1.5 | 0.6 | 2.43E-04 |
| 13 | 7 | 0 | 1.00E+18 | 1.5 | 0.8 | 2.92E-04 |
| 13 | 7 | 0 | 1.00E+18 | 1.5 | 1 | 3.18E-04 |
| 13 | 7 | 0 | 1.00E+18 | 1.5 | 1.2 | 3.30E-04 |
| 13 | 7 | 0 | 1.00E+18 | 1.5 | 1.4 | 3.35E-04 |
| 13 | 7 | 0 | 1.00E+18 | 1.5 | 1.6 | 3.38E-04 |
| 13 | 7 | 0 | 1.00E+18 | 1.5 | 1.8 | 3.41E-04 |
| 13 | 7 | 0 | 1.00E+18 | 1.5 | 2 | 3.43E-04 |
| 13 | 7 | 0 | 1.00E+18 | 1.5 | 2.2 | 3.45E-04 |
| 13 | 7 | 0 | 1.00E+18 | 1.5 | 2.4 | 3.47E-04 |
| 13 | 7 | 0 | 1.00E+18 | 1.5 | 2.6 | 3.48E-04 |
| 13 | 7 | 0 | 1.00E+18 | 1.5 | 2.8 | 3.50E-04 |
| 13 | 7 | 0 | 1.00E+18 | 1.5 | 3 | 3.51E-04 |
| 13 | 7 | 0 | 1.00E+18 | 1.5 | 3.2 | 3.53E-04 |
| 13 | 7 | 0 | 1.00E+18 | 1.5 | 3.4 | 3.54E-04 |
| 13 | 7 | 0 | 1.00E+18 | 1.5 | 3.6 | 3.56E-04 |
| 13 | 7 | 0 | 1.00E+18 | 1.5 | 3.8 | 3.57E-04 |
| 13 | 7 | 0 | 1.00E+18 | 1.5 | 4 | 3.58E-04 |
| 13 | 7 | 0 | 1.00E+19 | 1.5 | 0 | -9.99E-09 |
| 13 | 7 | 0 | 1.00E+19 | 1.5 | 0.2 | 4.58E-04 |
| 13 | 7 | 0 | 1.00E+19 | 1.5 | 0.4 | 7.44E-04 |
| 13 | 7 | 0 | 1.00E+19 | 1.5 | 0.6 | 8.56E-04 |
| 13 | 7 | 0 | 1.00E+19 | 1.5 | 0.8 | 8.88E-04 |
| 13 | 7 | 0 | 1.00E+19 | 1.5 | 1 | 9.05E-04 |
| 13 | 7 | 0 | 1.00E+19 | 1.5 | 1.2 | 9.19E-04 |

**Fig. S1: Labelled dataset for ML algorithm.**
